# Bottlenose dolphin habitat and management factors related to activity and distance traveled in zoos and aquariums

**DOI:** 10.1371/journal.pone.0250687

**Published:** 2021-08-30

**Authors:** Lisa K. Lauderdale, K. Alex Shorter, Ding Zhang, Joaquin Gabaldon, Jill D. Mellen, Michael T. Walsh, Douglas A. Granger, Lance J. Miller

**Affiliations:** 1 Conservation Science and Animal Welfare Research, Chicago Zoological Society – Brookfield Zoo, Brookfield, Illinois, United States of America; 2 Department of Mechanical Engineering, University of Michigan, Ann Arbor, Michigan, United States of America; 3 Robotics Institute, University of Michigan, Ann Arbor, Michigan, United States of America; 4 Biology Department, Portland State University, Portland, Oregon, United States of America; 5 Department of Comparative, Diagnostic & Population Medicine, College of Veterinary Medicine, University of Florida, Gainesville, Florida, United States of America; 6 Institute for Interdisciplinary Salivary Bioscience Research, University of California, Irvine, California, United States of America; Institute of Deep-sea Science and Engineering, Chinese Academy of Sciences, CHINA

## Abstract

High-resolution non-invasive cetacean tagging systems can be used to investigate the influence of habitat characteristics and management factors on behavior by quantifying activity levels and distance traveled by bottlenose dolphins (*Tursiops truncatus* and *Tursiops aduncus*) in accredited zoos and aquariums. Movement Tags (MTags), a bio-logging device, were used to record a suite of kinematic and environmental information outside of formal training sessions as part of a larger study titled “Towards understanding the welfare of cetaceans in zoos and aquariums” (colloquially called the Cetacean Welfare Study). The purpose of the present study was to explore if and how habitat characteristics, environmental enrichment programs, and training programs were related to the distance traveled and energy expenditure of dolphins in accredited zoos and aquariums. Bottlenose dolphins in accredited zoos and aquariums wore MTags one day per week for two five-week data collection periods. Overall dynamic body acceleration (ODBA), a proxy for energy expenditure, and average distance traveled per hour (ADT) of 60 dolphins in 31 habitats were examined in relation to demographic, habitat, and management factors. Participating facilities were accredited by the Alliance for Marine Mammal Parks and/or Aquariums and the Association of Zoos & Aquariums. Two factors were found to be related to ADT while six factors were associated with ODBA. The results showed that enrichment programs were strongly related to both ODBA and ADT. Scheduling predictable training session times was also positively associated with ADT. The findings suggested that habitat characteristics had a relatively weak association with ODBA and were not related to ADT. In combination, the results suggested that management practices were more strongly related to activity levels than habitat characteristics.

## Introduction

Bio-logging devices are tools which can be used to study animal kinematics and to monitor behavior. Bio-logging refers to the practice of attaching a miniaturized device that records and stores data related to an animal’s movement, behavior, physiology, and/or environment [1]. Bio-logging devices have been used to study the behavior and welfare of domesticated and non-domesticated animals in the wild as well as in farm, zoo, and aquarium environments. In domesticated species, they have been used to predict estrus in cows, quantify grazing patterns in cattle, identify heat stress indicators for swine, and examine welfare in both cattle and pigs [2–5]. In non-domesticated species, they have been used to study recumbency in giraffes under professional care [6] and they have also been used to study the migration patterns [7], nesting behaviors [8], and energy expenditure [9] of wild animals. Wearable tracking systems are an especially valuable tool for studying animals that are often out of view of direct observation such as wild cetaceans that spend their lives underwater and where sediment and algae can occlude them from view [10]. For cetaceans, bio-logging devices provide researchers a means of quantifying the impact of human activities [11–13], tracking behavior change [14–16], locating animals within the habitat [17, 18], capturing acoustics simultaneously with dynamic movement [19], and exploring diving behavior and fine-scale kinematics [20, 21].

High-resolution cetacean tagging systems tend to be non-invasive (e.g., attached via specially designed suction cups for cetaceans), and are designed to limit their effect on the animal’s hydrodynamics [22, 23]. These devices host a suite of onboard sensors that directly measure various kinematic and environmental data including accelerometers, magnetometers, gyroscopes, hydrophones, pressure sensors, thermometers, and global positioning systems. Parameters such as speed, acceleration, depth, diving behavior, and location can be derived from these sensors. The fine-scale data collected from these devices facilitate the investigation of fundamental questions in behavior, biology, and ecology [20, 24–26].

Bio-logging devices have been used broadly to study wild diving mammals [27] but their adoption for research on cetaceans within zoos and aquariums has been limited. Bottlenose dolphins (*Tursiops truncatus*) have been used to collect biomechanical data during prescribed tasks to characterize the effect of drag from the device on their metabolic rate, stroking effort, and swim speed [26, 28]. Shorter et al. [14] provides an account of an analysis of diving behavior and activity level of bottlenose dolphins under professional care. Using the bio-logging devices, they were able to detect day-scale changes in behavior. Consistent with studies using behavioral observation [29], the dolphins exhibited lower activity levels at night. In addition, they were able to establish time budgets for swimming types and characterize diving patterns.

Bio-logging tags are useful tools designed to quantify and monitor activity levels, which is an important indicator of welfare in combination with other physical and behavioral measures. Activity levels and locomotion are affected by various types of habitats and environmental enrichment and are used as an indicator of animal welfare [30–34]. Activity level increases and decreases may be a contributor to and an indicator of the physical health of an individual. Providing opportunities to exercise (i.e., active behaviors) can reduce the risk of developing illnesses and mitigate complications of current illnesses in several species [35–38]. In contrast, an extremely low activity level (i.e., lethargy) is considered an indicator of poor welfare [39–41]. In addition, locomotion, postural, and gait analyses have been used in welfare assessments to identify movement issues or lameness [42–44]. Bio-logging data has been used to identify specific behaviors, track activity levels over time, create activity budgets, and assess gait patterns [45–48]. Bio-logging tags are helpful tools in examining activity and locomotion because they generate fine-scale data on the movement of an individual. While gait has yet to be used in health assessments in dolphins, recent research has begun to establish bio-logging methods to quantify swimming patterns of bottlenose dolphins [14].

While these studies demonstrate the potential uses of bio-logging data by informing our understanding of dolphins under professional care, previous research has focused on a limited number of dolphins within the same habitat. However, dolphins under professional care live in habitats with a variety of features. They may be professionally managed zoo/aquarium habitats or professionally managed ocean habitats, consist of a single area or be comprised of multiple gated environments, and vary in depth, length, width, and shape. A comparative examination of how different environments may be related to activity is an open question. The purpose of the present study was to use data from a bio-logging device to explore if and how habitat characteristics, environmental enrichment programs, and training programs were related to the distance traveled and energy expenditure of dolphins in accredited zoos and aquariums.

## Materials and methods

### Ethics statement

This study was authorized by the management at each participating zoo and aquarium and, where applicable, was reviewed and approved by research committees. In addition, the study protocol was reviewed and approved by the U.S. Navy Marine Mammal Program Institutional Animal Care and Use Committee #123–2017.

### Subjects and facilities

This study is part of a larger project entitled “Towards Understanding the Welfare of Cetaceans in Zoos and Aquariums” (colloquially called the Cetacean Welfare Study). Zoos and aquariums that were accredited in 2017 by the Alliance for Marine Mammal Parks and Aquariums and the Association of Zoos & Aquariums were eligible for participation in this portion of the larger Cetacean Welfare Study provided they cared for common bottlenose dolphins or Indo-Pacific bottlenose dolphins (*Tursiops aduncus*). Two animals from each participating facility were selected using a semi-random sampling design in order to create a balanced representation of the study population. In total, data were collected from 65 dolphins at 35 habitats. Participating habitats were located in Bermuda (n = 1), Hong Kong (n = 1), Jamaica (n = 2), Mexico (n = 15), Singapore (n = 1), Spain (n = 1), and the United States (n = 14). Information on the full dataset is given in S1 Appendix including sex, age, and total minutes recorded outside of formal training sessions.

### Independent variables

Independent variables were selected to examine a range of habitat characteristics and management factors that could impact animal welfare. Independent variables and definitions are listed in Table 1. Definitions of terms and methods for calculating the synthesized independent variables as well as enrichment types are presented in Lauderdale et al [49].

**Table 1:** Independent variables included in the analysis.

Variable	Definition	Values	Type of Variable
***Demographic***			
Sex	Sex of the dolphin	Male/Female	Factor
Age	Age of the dolphin	Years	Covariate
***Environmental Enrichment***			
Enrichment Diversity Index	Enrichment diversity index was created using the Shannon diversity index on the mean number of days each enrichment is provided at the facility	Index	Covariate
Enrichment Program Index	Enrichment program index is a standardized factor score created with scores on frequency of enrichment program components used at the facility using a polychoric PCA	Index	Covariate
Night Time Enrichment	Mean number of nights in a week that enrichment was provided to the dolphins at the facility	Number of Nights	Covariate
Enrichment Schedule	Categorical value indicating how enrichment was scheduled at the facility	Predictable, Semi-Random, Random	Factor
Frequency of New Enrichment	Categorical frequency that a facility provided the dolphins with new types/forms of enrichment	Weekly/Monthly, Twice a Year, Yearly/Year+	Factor
***Training***			
Dolphin Presentations	Mean number of dolphin presentations an individual dolphin participated in each week	Mean Number of Presentations	Covariate
Interaction Programs	Mean number of dolphin interaction programs an individual dolphin participated in each week	Mean Number of Interactions	Covariate
Training Duration	Mean amount of time each dolphin interacted with an animal care professional for presentations, interaction programs, training sessions, research, or other training activities each week	Hours	Covariate
Maximum Number of Interaction Guests	Maximum number of participants allowed for an interaction program for that facility	Number of Participants	Covariate
Training Schedule	Categorical variable indicating if the training schedule for the dolphins at that facility was predictable or semi-predictable	Predictable, Semi-Predictable	Factor
***Habitat Characteristics***			
Day Time Spatial Experience	Proportionate volume of water the dolphin had access to based on the percentage of daytime hours spent in different habitats in each five-week data collection period	Megaliter	Covariate
Night Time Spatial Experience	Proportionate volume of water the dolphin had access to based on the percentage of night time hours spent in different habitats in each five-week data collection period	Megaliter	Covariate
24 Hour Spatial Experience	Proportionate volume of water the dolphin had access to based on the percentage of hours throughout the entire day spent in different habitats in each five-week data collection period	Megaliter	Covariate
Length	The maximum straight length in any direction across any habitat the dolphin had access to in each five-week data collection period	m	Covariate
Depth	The maximum depth for any habitat the dolphin had access to in each five-week data collection period	m	Covariate
Habitat Type	Categorical variable indicating the dolphin was in a professionally managed zoo/aquarium habitat or a professionally managed ocean habitat	Zoo/Aquarium, Ocean	Factor
Number of Habitats	Maximum number of habitats (different enclosures) dolphin had access to in daytime hours during each five-week data collection period	Number of Habitats	Covariate
Social Management	Categorical variable indicating the type of social management practice for a dolphin during each five-week data collection period	Same Group, Split/Reunited, Rotated Subgroups	Factor
Neighboring Conspecifics	Categorical variable indicating if the dolphin had visual and auditory access to other dolphins without possibility of physical contact during each five-week data collection period	No, Yes	Factor

### Data collection

**Fig 1:**
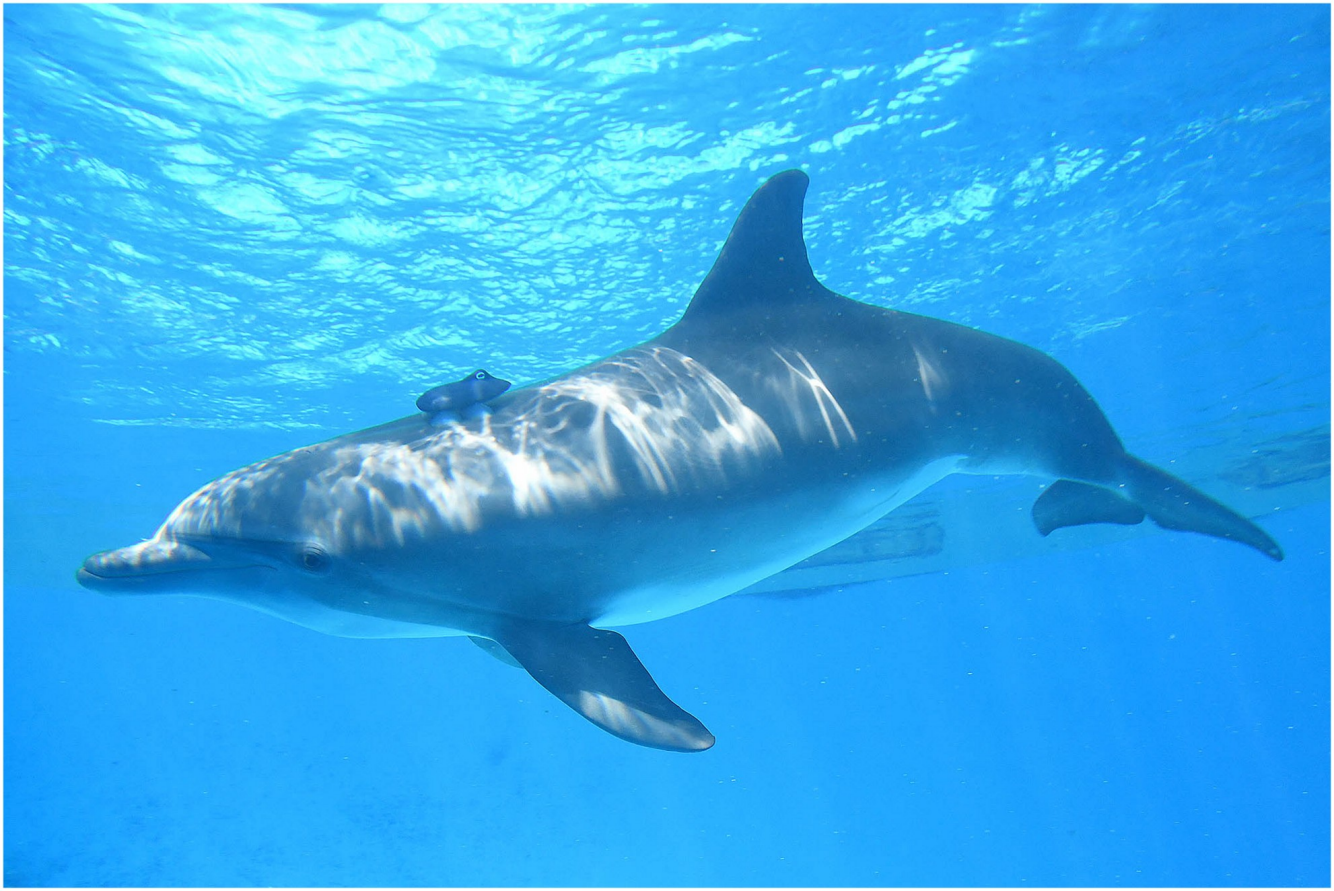
A bottlenose dolphin wearing an MTag. The MTag was secured to the dolphin approximately 20 cm behind the blowhole using four suction cups.

**Fig 2:**
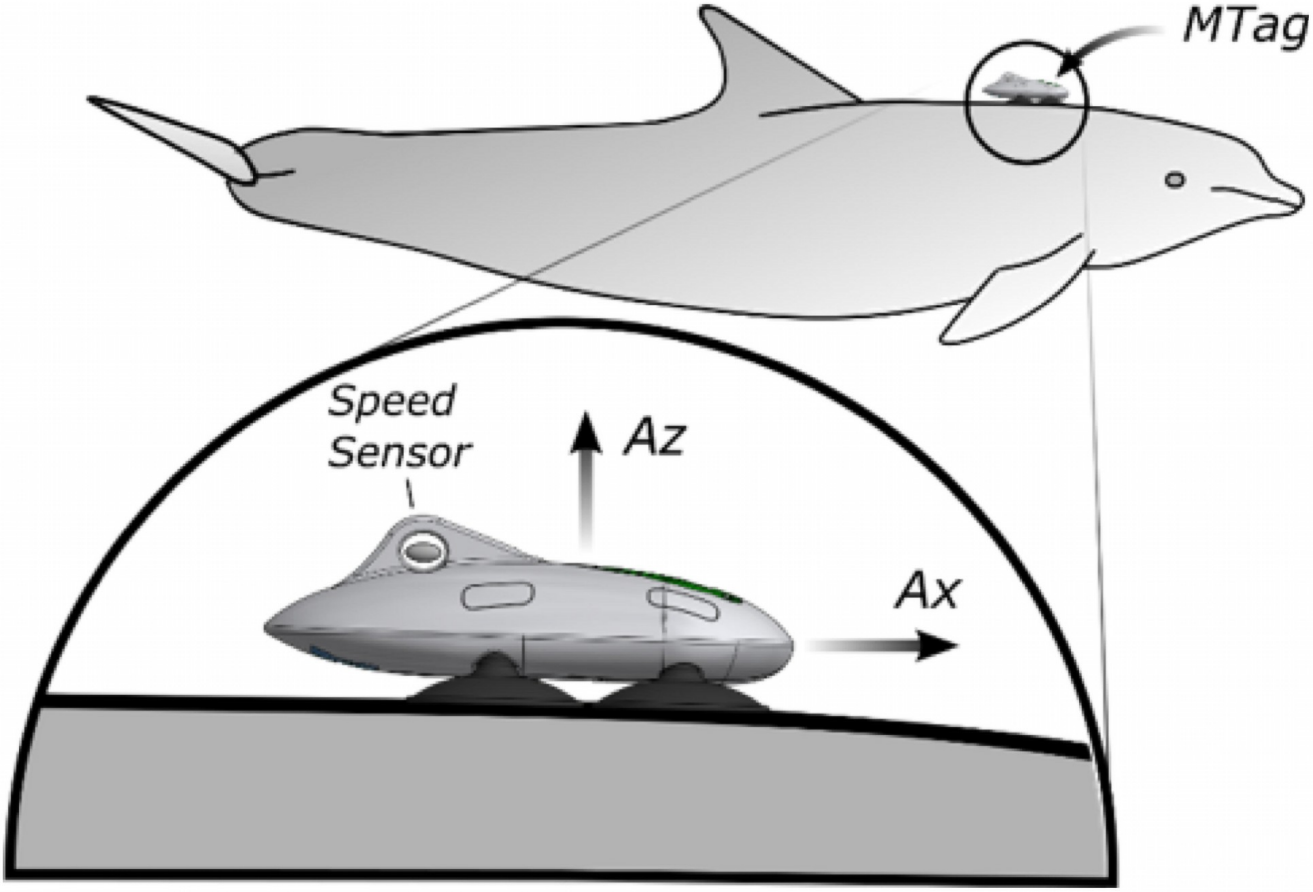
An illustration of the MTag. Speed through the water was measured with an externally mounted speed sensor, and acceleration was measured with an internal 3-axis accelerometer.

The Movement Tags (MTags) used in the study were 150 mm in length and 76 mm wide with four soft silicone cups (Figs 1 and 2). The primary housing of the MTag was 3D-printed using the FormLabs Form 2^™^ stereolithography process. The internal electronics were based on the Loggerhead Instruments OpenTag3 board. The OpenTag3 contained a nine degree-of-freedom (DOF) inertial measurement unit (IMU) with an accelerometer, gyroscope, and magnetometer. On-board sensors also recorded environmental temperature and pressure. Speed of the animal through water was measured using a magnetic micro-turbine mounted outside the tag housing and a 1-DOF Hall effect sensor (Fig 2). The IMU data from the accelerometer, gyroscope, and magnetometer were recorded at sampling rate of 50 Hz (i.e., samples per second) and the remaining sensors were recorded at a sampling rate of 5 Hz. The MTag was attached dorsally to the dolphin approximately 20 cm behind the blowhole with four silicone suction cups designed specifically for cetacean skin. Dolphins were trained to wear the MTags prior to the study in order to habituate the focal dolphin and their conspecifics to its presence. The MTags could be easily removed by animal care staff at any time. The MTags did not result in any damage to the skin and similar bio-logging devices have been used extensively with wild dolphins prior to application in this study.

Data were collected during five-week periods from July 2018 through November 2018 and from January through April 2019. MTags were deployed Tuesdays and Fridays using an alternating schedule for the two participants at each location. Each dolphin wore the MTag throughout its normal daily activities once a week.

### Data analysis

**Fig 3:**
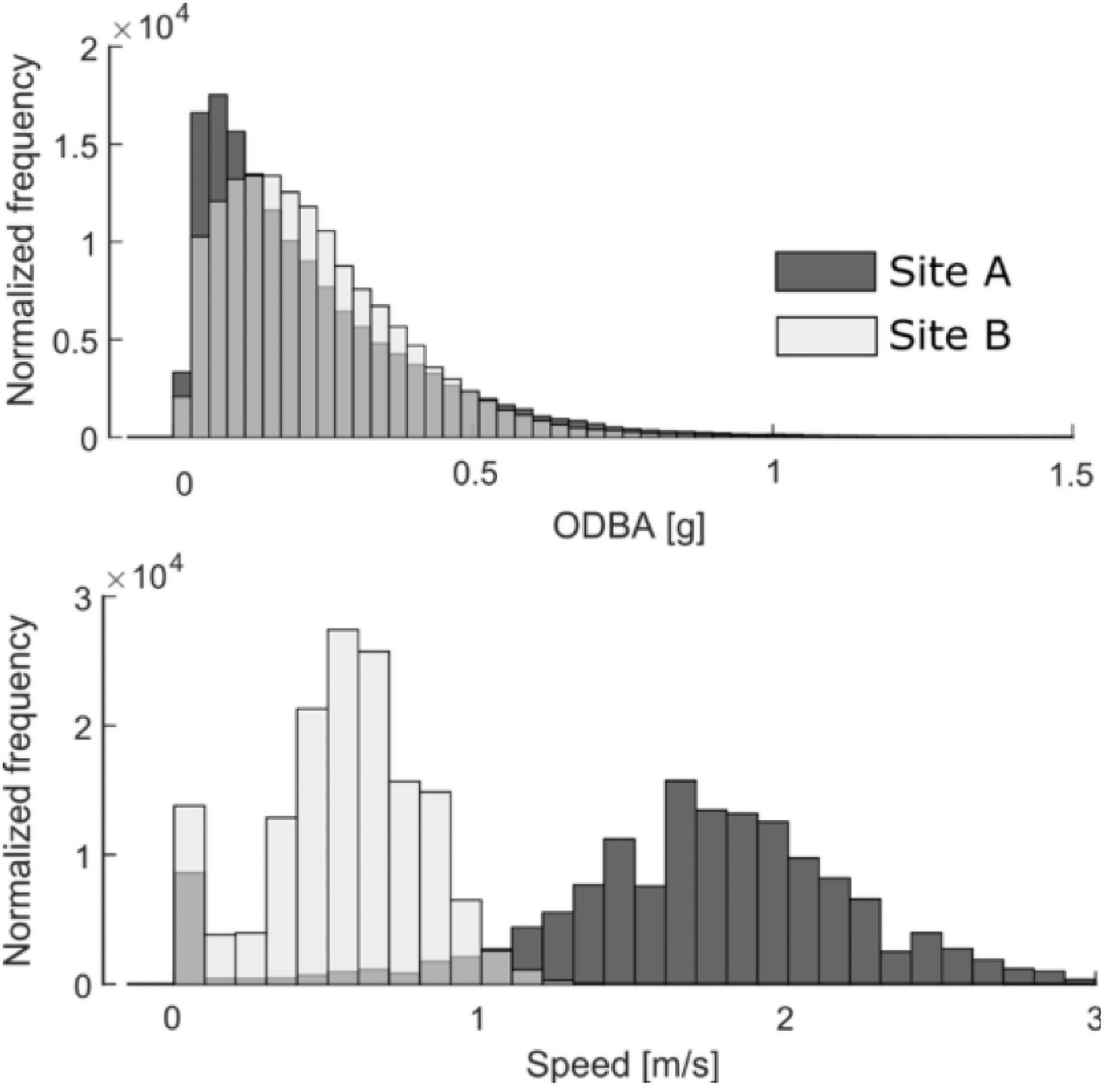
Representative summary measures of speed and ODBA. Data was taken from two dolphins at different facilities. A two second moving average was used to calculate the parameters from ~1 hr of data from each dolphin.

**Fig 4:**
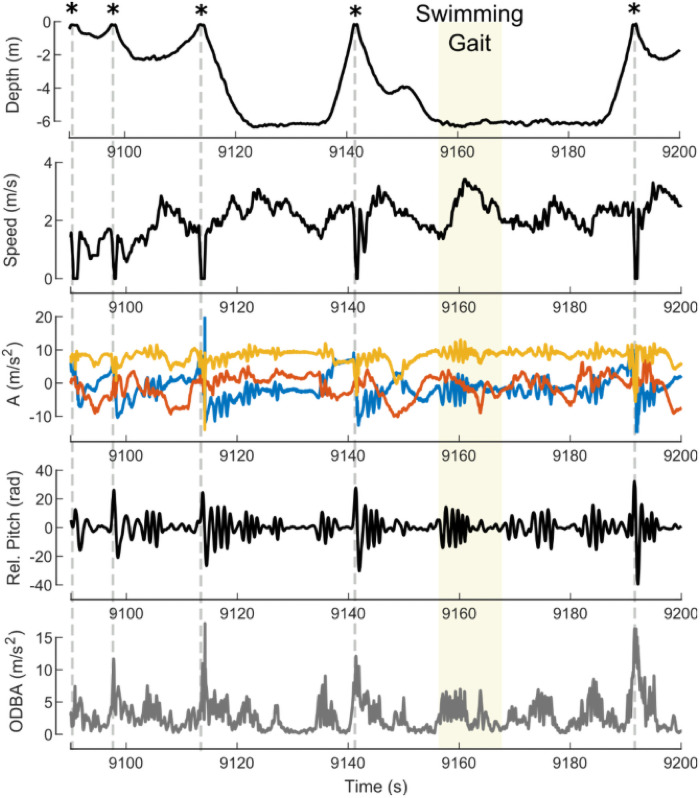
A representative section of swimming data. Surfacings are indicated with (*), and an example bought of swimming is highlighted. During this example, the animal uses a fluke and glide gait to move through the water.

Data from the accelerometer, gyroscope, and magnetometer on the MTags were used to estimate pitch, roll, and heading [50]. Relative pitch was calculated from the original pitch data by subtracting the low passed filtered static pitch component using a moving average filter with a window size of 1.5 seconds. Specific acceleration was calculated using information about the orientation of the MTag to subtract the gravitational acceleration measured by the accelerometers. To measure speed through water, the revolution rate of the micro-turbine sensor on the MTag was converted to speed through a linear calibration (slope-intercept). The calibration and verification for this speed sensor configuration is detailed in Gabaldon et al [51]. Measurement of speed through water was numerically integrated to estimate total distance traveled by the animal while wearing the tag. Overall dynamic body acceleration (ODBA) was also calculated from the accelerometer data, and was used to parameterize relative activity of the animal [52]. To quantify activity, a moving average with a two-second window was used on the ODBA values (Fig 3). A representative time series plot of these measurements and metrics is presented in Fig 4. Analyses were conducted in MATLAB 9.7.0 using custom scripts.

### Statistical analysis

MTag data were selected during times in which dolphins were outside of formal training sessions in order to explore dolphin behavior throughout the day. Demographic and management characteristics were evaluated for their association with ODBA and average distance traveled per hour (ADT). The distance was divided by the number of minutes recorded in the section and converted to a per hour rate. Statistical models were examined using generalized estimating equations (GEE) due to the non-normal distribution of the data. GEEs do not require data to be transformed which can preserve interpretability of the results [53, 54].

In addition to wearing the bio-logging devices, the dolphins were video recorded three times per week for 25 minutes over the course of the five-week period [55]. The minimum data criteria to be included in that study were that each dolphin would have at least 10 video observations and could be seen for the majority of those observations. This resulted in the 240-minute minimum criteria. The same criteria were used for the present study to remain consistent across manuscripts within the collection. Individuals with less than 240 minutes of MTag data recorded outside of formal training sessions in the 2018 and 2019 data collection periods were excluded from the analysis. If a dolphin had more than 240 minutes of data recorded in both the 2018 and 2019 data collection period, the 2018 data were used for analysis and the 2019 data were excluded. If only one of the two data collection periods had more than 240 minutes, the period with more than 240 minutes was retained for analysis and the other period was excluded. Data from a single five-week period were used due to the way GEEs handle missing data. Dolphins without data for both 2018 and 2019 would be entirely excluded from the analysis. These decisions were made to retain the robust sample size while ensuring the validity of the results. The goal was to prioritize an investigation of variability across accredited facilities rather than examining within subject variability. A chi-square test of significance and an independent t-test were used to determine if the age and sex demographics of the dolphins in the final data set were statistically different than the original group of dolphins sampled.

Predictive regression models were fitted using GEEs to allow for individual level analysis and to account for facility ID. Facility ID was considered a random effect with an independent correlation structure. Regression models were built first with univariate level predictors. Predictor variables with sample sizes less than three were not considered for further analysis. Predictor variables correlating (*p* < 0.15) with the dependent variable were retained for evaluation in building the multivariate models. The final multivariate model was selected based on the quasi-likelihood under the independence (QIC) value and the number of significant independent variables. Statistical analyses for the GEE regression models were conducted in SPSS 21. The final models that were considered with significant independent variables and the lowest QIC values are given in S1 File.

## Results

Sixty dolphins in 31 habitats met the minimum criteria for inclusion based on minutes of data collected outside of formal training sessions. The sex (χ^2^(1, N = 125) = 3.623, *p* = 0.057) and age (*t*(123) = 0.542, *p* = 0.589) distribution of the group of participants included in the analyses were not significantly different than the group composition prior to excluding dolphins that did not meet the inclusion criteria. Of these, 57 individuals were common bottlenose dolphins and three were Indo-Pacific bottlenose dolphins. Dolphins that met the minimum criteria for inclusion ranged in age from 3 to 44 years at the start of data collection (mean 16.48 ± 9.84 SD). In total, 1053.35 hours of data (range: 255 to 2043 minutes per dolphin) were collected during periods in which the dolphin was outside of formal training sessions. On average, dolphins traveled 2.32 ± 1.13 km per hr. The mean ODBA value for all participants was 2.31 ± 0.63 m/s^2^. The mean maximum depth was 5.63 m for the professionally managed ocean habitats and 8.78 m for professionally managed zoo/aquarium habitats.

Demographic and management factors were evaluated in relation to ODBA and ADT. Univariate correlations where *p* < 0.15 were observed between ODBA and two demographic variables, three enrichment variables, two training variables, and seven habitat variables (Table 2). Univariate correlations where *p* < 0.15 were observed between ADT and one enrichment variable, one training variable, and seven habitat variables (Table 3). Descriptive statistics for independent variables included in the multivariate modeling process are presented in Table 4.

**Table 2:** Univariate correlations between ODBA and independent variables.

Variables	Reference	n	Beta	*p*-value
***Demographic***				
Sex	Ref ^a^ = Male	35	0.000	
	Female	25	0.372	0.020*
Age		60	-0.027	0.003*
***Environmental Enrichment***				
Enrichment Diversity Index		60	0.031	0.800
Enrichment Program Index		60	0.022	0.741
Night Time Enrichment		60	-0.059	0.073^
Enrichment Schedule	Ref = Predictable	8	0.000	
	Semi-Random	44	0.272	0.018*
	Random	8	-4.320	0.015*
Frequency of New Enrichment	Ref = Year+	2	0.000	
	Twice a Year	16	-0.145	0.213
	Monthly/ Weekly	42	0.246	0.029*
***Training***				
Dolphin Presentations		60	0.012	0.185
Interaction Programs		60	0.008	0.527
Training Duration		60	0.033	0.000*
Maximum Number Interaction Guests		60	0.009	0.356
Training Schedule	Ref = Predictable	29	0.000	
	Semi-Predictable	31	-0.391	0.013*
***Habitat Characteristics***				
Day Time Spatial Experience		60	0.049	0.000*
Night Time Spatial Experience		60	0.047	0.000*
24 Hour Spatial Experience		60	0.049	0.000*
Length		60	0.007	0.049*
Depth		60	-0.044	0.005*
Habitat Type	Ref = Zoo/Aquarium Habitat	35	0.000	
	Ocean Habitat	25	0.244	0.112^
Number of Habitats		60	-0.087	0.018*
Social Management	Ref = Same Group	29	0.000	
	Split/Reunited at Night	20	0.100	0.575
	Rotated Subgroups	11	-0.226	0.295
Neighboring Conspecifics	Ref = No Visual Access	35	0.000	
	Visual/Auditory Access	25	-0.212	0.186

^^^*p*-value < 0.15 used as threshold significant level for model building

*p-value < 0.05

^a^ The reference value (Ref =) was the baseline value used when calculating univariate correlations with these binary variables.

**Table 3:** Univariate correlations between distance traveled and independent variables.

Variables	Reference	n	Beta	*p*-value
***Demographic***				
Sex	Ref ^a^ = Male	35	0.000	
	Female	25	0.281	0.311
Age		60	0.008	0.596
***Environmental Enrichment***				
Enrichment Diversity Index		60	-0.177	0.485
Enrichment Program Index		60	0.018	0.901
Night Time Enrichment		60	-0.026	0.621
Enrichment Schedule	Ref = Predictable	8	0.000	
	Semi-Random	44	0.494	0.203
	Random	8	-0.307	0.462
Frequency of New Enrichment	Ref = Year+ / Yearly	2	0.000	
	Twice a Year	16	0.347	0.158
	Monthly / Weekly	42	0.946	<0.001*
***Training***				
Dolphin Presentations		60	0.001	0.909
Interaction Programs		60	0.012	0.553
Training Duration		60	-0.007	0.632
Maximum Number Interaction Guests		60	0.014	0.336
Training Schedule	Ref = Predictable	29	0.000	
	Semi-Predictable	31	-0.697	0.012*
***Habitat Characteristics***				
Day Time Spatial Experience		60	-0.003	0.852
Night Time Spatial Experience		60	-0.020	0.244
24 Hour Spatial Experience		60	-0.011	0.467
Length		60	-0.002	0.608
Depth		60	-0.004	0.878
Habitat Type	Ref = Zoo/Aquarium Habitat	35	0.000	
	Ocean Habitat	25	0.077	0.796
Number of Habitats		60	0.024	0.684
Social Management	Ref = Same Group	29	0.000	
	Split/Reunited at Night	20	0.528	0.095^
	Rotated Subgroups	11	0.342	0.402
Neighboring Conspecifics	Ref = No Visual Access	35	0.000	
	Visual/Auditory Access	25	-0.504	0.074^

^^^*p*-value < 0.15 used as threshold significant level for model building

*p-value < 0.05

^a^ The reference value (Ref =) was the baseline value used when calculating univariate correlations with these binary variables.

**Table 4:** Descriptive statistics for the independent variables included in the multivariate modeling process.

Independent Variable	Reference	n	Mean	SD	Min	Max	Median
Sex	Ref^a^ = Male	35	-	-	-	-	-
	Female	25	-	-	-	-	-
Age		60	16.48	9.84	3.00	44.00	14.00
Enrichment Schedule	Ref = Predictable	8	-	-	-	-	-
	Semi-Random	44	-	-	-	-	-
	Random	8	-	-	-	-	-
Frequency of New Enrichment	Ref = Year+ / Yearly	2	-	-	-	-	-
	Twice a Year	16	-	-	-	-	-
	Monthly / Weekly	42	-	-	-	-	-
Training Schedule	Ref = Predictable	29	-	-	-	-	-
	Semi-Predictable	31	-	-	-	-	-
Day Time Spatial Experience		60	2931.47	4361.90	38.12	25200.00	1700.00
Depth		60	7.47	4.39	3.00	18.11	5.00

^a^ The reference value (Ref =) was the baseline value used when calculating univariate correlations with these binary variables.

The final multivariate model for ODBA included sex, age, enrichment schedule, day time spatial experience, frequency of new enrichment, and depth (Table 5). ODBA was higher for females when compared to males (β = 0.32, *p* = 0.02). ODBA values decreased as the age of the dolphin increased (β = -0.02, *p* = 0.01; i.e., older dolphins had lower ODBA values) and dolphins provided with enrichment on a random schedule had lower ODBA values than those provided enrichment on a predictable schedule (β = -0.35, *p* = 0.03). ODBA values were higher for dolphins receiving new enrichment on a monthly/weekly schedule than dolphins receiving new enrichment on a yearly/year+ schedule (β = 0.30, *p* = 0.01). ODBA values increased as day time spatial experience increased (β = 0.03, *p* < 0.01). ODBA values decreased as the maximum depth of the habitat increased (β = -0.03, *p* = 0.05).

**Table 5:** Results for the full multivariate model examining ODBA.

Variable	Beta	Std error	Lower 95% CI	Upper 95% CI	*p*-value
(Intercept)	2.26	0.18	1.92	2.61	<0.01
Sex: Male	-	-	-	-	-
Sex: Female	0.32	0.13	0.06	0.58	0.02*
Age	-0.02	0.01	-0.04	-0.01	0.01*
Enrichment Schedule: Predictable	-	-	-	-	-
Enrichment Schedule: Semi-Random	0.19	0.13	-0.06	0.43	0.13
Enrichment Schedule: Random	-0.35	0.16	-0.67	-0.04	0.03*
Frequency of New Enrichment: Yearly / Year+	-	-	-	-	-
Frequency of New Enrichment: Twice per year	0.22	0.13	-0.03	0.48	0.09
Frequency of New Enrichment: Monthly / Weekly	0.30	0.12	0.07	0.53	0.01*
Day Time Spatial Experience	0.03	0.01	0.01	0.04	<0.01*
Depth	-0.03	0.01	-0.05	0.00	0.05*

*p-value < 0.05

The final multivariate model for ADT included the frequency of new enrichment and training schedule (Table 6). ADT values were higher for dolphins who received new enrichment on a twice per year schedule than dolphins who received new enrichment on a yearly/year+ schedule (β = 0.62, *p* < 0.01). ADT values were higher for dolphins participating in training sessions on semi-predictable schedules than those participating on predicable schedules (β = -0.56, *p* = 0.04). Descriptive statistics for independent variables included in the multivariate modeling process are presented in Table 4.

**Table 6:** Results for the full multivariate model examining average distance traveled per hour.

Variable	Beta	Std error	Lower 95% CI	Upper 95% CI	*p*-value
(Intercept)	2.73	0.23	2.29	3.18	<0.01
Frequency of New Enrichment: Yearly / Year+	-	-	-	-	-
Frequency of New Enrichment: Twice per year	0.62	0.20	0.22	1.02	<0.01*
Frequency of New Enrichment: Monthly / Weekly	0.21	0.27	-0.32	0.73	0.44
Training Schedule: Predictable	-	-	-	-	-
Training Schedule: Semi-Predicable	-0.56	0.27	-1.09	-0.02	0.04*

*p-value < 0.05

## Discussion

This research marks the first large scale study to investigate bottlenose dolphin activity and energy expenditure using high resolution wearable data tagging systems in accredited zoos and aquariums. The participants provided data from a multitude of habitats and individuals thereby enabling robust conclusions.

ODBA (i.e., overall dynamic body acceleration) and ADT (i.e., average distance traveled per hour) represent two metrics that can be used to quantify activity and characterize two ways in which bottlenose dolphins use their habitat. While ODBA is not a validated indicator of welfare, the results suggested that several demographic, enrichment, and habitat characteristic variables were related to ODBA. Two enrichment-related factors (i.e., enrichment schedule and frequency of new enrichment) played a significant role in our final model. Supplying novel enrichment on a monthly/weekly schedule were associated with higher ODBA values than on a yearly/year+ schedule. Dolphins receiving enrichment on a random schedule had lower ODBA values than those receiving enrichment on a predictable schedule. Predictability of favorable events is thought to be an important part of animal welfare [56]. Events can be predictable based on temporal schedules or by establishing signals that precede the event. Watters and colleagues [57] found that moderate predictability in food delivery times stimulated species-specific behaviors and reduced anticipatory behaviors when compared to completely predictable and completely unpredictable schedules. One possible reason for the finding that ODBA had a negative relationship with random enrichment schedules may be that dolphins remained near areas where enrichment is provided and were more vigilant in a nearby area than active throughout the habitat during those times. If this is behavior commonly seen by animal care staff with a specific group or individual, they could mitigate this by establishing a signal that precedes the addition of enrichment objects. Based on findings from other analyses from the Cetacean Welfare Study, group active behaviors and interacting with conspecifics were higher for dolphins receiving enrichment on a predictable schedule than those receiving enrichment on a random schedule [10]. Group active behaviors and interacting with conspecifics were also higher for dolphins receiving new enrichment on a monthly/weekly schedule and/or a biannual schedule when compared those receiving new enrichment on a yearly/year+ schedule [10, 55]. These findings suggested ODBA, ADT, and social behaviors may be intertwined.

Two space use factors were included in the final ODBA model. Access to larger volumes of water during the day were positively related to ODBA values, and deeper habitats were negatively related to ODBA values. Similarly, the data from the Cetacean Welfare Study also indicated that behavioral diversity decreased as the maximum depth of the habitat increased [58]. Importantly, ODBA values were derived from acceleration measurements, which are related to changes in the speed of the animal and other forces, like water impacting the MTag as it moves through the air/water interface while surfacing. More surfacing events during a given amount of time would increase the noise in the acceleration measurements masking the acceleration of the animal. However, deeper depths may have been associated with lower ODBA values because swimming at the bottom and ascent portion of a dive sequence may require less active swimming than what is required for multiple short surfacing events [14]. ODBA values are lowest during gliding portions of swimming gates and during ascent as dolphins use their positive buoyancy to reduce the need to actively fluke. The ability to dive deeper and utilize gliding patterns may result in a small reduction in ODBA when compared to periods of continuous fluking. Given the small beta coefficients for the relationship between day time spatial experience and ODBA, even substantial increases in space would yield only minimal increases in ODBA values. This suggested that larger volumes of water were not the primary driver of activity when compared to animal management factors.

Small decreases in ODBA were associated with increased age (i.e., older dolphins). This is consistent with previous reports that high energy activities such as play are more common in younger dolphins [59]. As age increased, use of the top third of the habitat decreased [60] suggesting that older dolphins may use the bottom of the habitat for solitary or group swimming. Females were found to have significantly higher ODBA values than males across facilities. This finding has not been previously reported and both females and males are generally considered to be highly social [61, 62]. As such, this is an important avenue of future research. While day time spatial experience, depth, and age were included in the ODBA final model, the associations were weak based on the beta coefficients when compared to sex and the enrichment variables. Thus, the frequency with which new environmental enrichment was given and the schedule it was supplied on had a stronger relationship to activity levels than the size of the habitat.

The dolphins in the present study traveled an average of 2.32 km per hr. Previous research has shown dolphins in Sarasota Bay, Florida swim at a mean speed of approximately 1.2 km per hour during the day [63]. Wild bottlenose dolphins on the Pacific Coast of the United States have been recorded swimming between 11 and 47 km over a 24 hour period [64]. This would equate to between 0.46 km per hr and 1.96 km per hr which are well below the rate found in the current study. While data for this work were collected only during daytime hours, it has been reported that wild bottlenose dolphins are active throughout the night whereas dolphins under human care reduce their activity comparatively [65, 66]. Prior reports suggest that dolphins under human care have reduced levels of activity during nighttime hours [14, 29]. It is possible this pattern is related to the fact that training sessions are generally concentrated during daytime hours. Future research should examine the influence of the number and spacing of training sessions (i.e., when the dolphins receive the majority of their diet) on ODBA and ADT.

Previous research has found that dolphins spent more time floating when in a smaller, zoo/aquarium habitat than in a larger ocean habitat during the day [67, 68]. However, habitat characteristics were not found to be related to ADT in the present study. The final multivariate model for ADT included the frequency of new enrichment and the training schedule. Supplying new environmental enrichment items twice per year was associated with higher ADT. Novelty and resistance to habituation are important factors to consider when implementing an enrichment program [69]. Semi-predictable training schedules were associated with lower distance traveled values when compared to predictable schedules. One hypothesis is that predictable training schedules allowed dolphins to express specific behaviors at appropriate times outside of formal training sessions in relation to the schedule. In addition, other findings from the Cetacean Welfare Study suggested that behavioral diversity was higher for dolphins participating in training sessions on a semi-predictable training schedule than those participating in training sessions on a random schedule [55]. Semi-predictable training schedules may result in dolphins maintaining vigilance in anticipation of the next session thereby reducing the distance they swim outside of formal training sessions.

Limitations of the study include the potential effects of wearing the MTags. While dolphins were trained and habituated to wearing the MTags prior to the onset of data collection, it is possible that the dolphin participants or their conspecifics were modifying their behavior due to its presence. Additionally, the MTag adds drag to dolphin’s fusiform body and may have resulted in changes in behavior. DTAGs, a larger but similarly shaped tag, do not affect bottlenose dolphins oxygen consumption, physical activity ratios, cost of transport, or locomotor costs during structured swimming tasks [26]. However, the DTAGs may have affected swimming speed as dolphins swam significantly slower (11%) when wearing DTAGs during the structured swimming task in that study [26]. It is possible that dolphins in the present study also slowed their swimming in order to compensate for the MTag despite its smaller size. Additionally, the ADT values may be an underestimate to the actual distance traveled. This parameter was recorded using a micro-turbine mounted on top of the MTag that spun as the dolphins swam through the water. Thus, when the dolphin is swimming at the surface of the water, the turbine is not spinning and recording revolutions. Dolphins who spent larger portions of time with the MTag above water would result in artificially lower ADT values.

Other factors that may influence ODBA and ADT include the animals diet, training schedule, social composition, and dominance structure of the group. For example, dominant individuals might influence the speed and directionality of the group. The results may also be limited by the inclusion of both *Tursiops aduncus* and *Tursiops truncatus*. Future research should investigate potential differences in activity levels between the subspecies while under professional care. Another limitation was data loss due to water damage to some of the MTags. Several dolphins were removed from the study because the MTags were water damaged before they met the minimum inclusion criteria. MTags are a relatively new technology and MTag deployments on this scale are unprecedented. Thus, it was unsurprising that, on occasion, a device would be damaged due to the closure system that protected the internal components. Future designs of the MTag will operate wirelessly to remove the possibility of the internal components receiving water damage.

The goal of the present study was to use bio-logging tags to investigate if and how habitat characteristics and management factors were related to the activity levels and distanced traveled by bottlenose dolphins in accredited zoos and aquariums. The results showed that enrichment programs played a significant role in both ODBA and ADT. Scheduling predictable training session times was also associated with higher ADT. The findings suggested that habitat characteristics had a relatively weak association with ODBA and were not related to ADT. In combination, the results suggested that management practices were more influential on activity levels than habitat characteristics. These data revealed important relationships between dolphins’ swimming behavior and a facility’s management practices as well as habitat characteristics. These findings demonstrated that the data obtained by bio-logging devices has the potential to inform managers and veterinarians of changes in health and welfare in real time, allowing for rapid veterinary and behavioral interventions aimed at promoting optimal health and welfare. Future research should investigate the types of activities and swimming styles in relation to normal behavior.

## Supporting information

S1 Appendix Lauderdale_ Activity. (XLSX)

S1. File ModelSelection_Lauderdale_Activity. (XLSX)

S2. File DescriptiveStatistics_Lauderdale_Activity. (XLSX)
